# African mitochondrial haplogroup L7: a 100,000-year-old maternal human lineage discovered through reassessment and new sequencing

**DOI:** 10.1038/s41598-022-13856-0

**Published:** 2022-06-24

**Authors:** Paul A. Maier, Göran Runfeldt, Roberta J. Estes, Miguel G. Vilar

**Affiliations:** 1 FamilyTreeDNA, Gene By Gene, 1445 N Loop W, Houston, TX 77008 USA; 2 Department of Evolution, Ecology, and Organismal Biology, University of California Riverside, 900 University Ave, Riverside, CA 92521 USA; 3 DNAeXplained, Brighton, MI 48114 USA; 4 Department of Anthropology, University of Maryland, 7999 Regents Dr, College Park, MD 20740 USA; 5 Genographic Project, National Geographic Society, 1145 17th St NW, Washington, DC 20036 USA; 6 Million Mito Project, Houston, TX 77008 USA

**Keywords:** Population genetics, Phylogenetics, Biological anthropology

## Abstract

Archaeological and genomic evidence suggest that modern *Homo sapiens* have roamed the planet for some 300–500 thousand years. In contrast, global human mitochondrial (mtDNA) diversity coalesces to one African female ancestor (“Mitochondrial Eve”) some 145 thousand years ago, owing to the ¼ gene pool size of our matrilineally inherited haploid genome. Therefore, most of human prehistory was spent in Africa where early ancestors of Southern African Khoisan and Central African rainforest hunter-gatherers (RFHGs) segregated into smaller groups. Their subdivisions followed climatic oscillations, new modes of subsistence, local adaptations, and cultural-linguistic differences, all prior to their exodus out of Africa. Seven African mtDNA haplogroups (L0–L6) traditionally captured this ancient structure—these L haplogroups have formed the backbone of the mtDNA tree for nearly two decades. Here we describe L7, an eighth haplogroup that we estimate to be ~ 100 thousand years old and which has been previously misclassified in the literature. In addition, L7 has a phylogenetic sublineage L7a*, the oldest singleton branch in the human mtDNA tree (~ 80 thousand years). We found that L7 and its sister group L5 are both low-frequency relics centered around East Africa, but in different populations (L7: Sandawe; L5: Mbuti). Although three small subclades of African foragers hint at the population origins of L5'7, the majority of subclades are divided into Afro-Asiatic and eastern Bantu groups, indicative of more recent admixture. A regular re-estimation of the entire mtDNA haplotype tree is needed to ensure correct cladistic placement of new samples in the future.

## Introduction

Descent with modification^1^ is perhaps the greatest insight in all biology. By inferring common ancestry between orthologous DNA sequences, the field of molecular phylogenetics has revolutionized how we design cancer therapies^2^, trace infectious diseases^3^, unlock the secrets of aging^4^, and ultimately study human history^5^. Mitochondrial DNA (mtDNA) is the oldest and most ubiquitous genetic tool for revealing the shared origins and migration patterns of humankind^6–8^. Its uniparental inheritance and unique properties make it ideal for reconstructing the human matrilineal phylogeny: abundant cytoplasmic copies make it easy to sequence, rapidly accumulating mutations can resolve recent events, and non-recombining transmission allows for ancient relationships to be finely estimated.

Since the original Cambridge Reference Sequence was published 40 years ago^9^, the field of human mtDNA phylogenetics has increasingly shown that non-African lineages are a subset, or small clade nested within a much older and hyper-diverse African phylogeny^6,8,10,11^. That same pattern of ancient African origins and subsequent Out-of-Africa (OOA) expansion some 70–50 thousand years ago (kya) has since been corroborated by Y-DNA^12,13^, autosomal DNA^14–16^, patterns of archaic introgression^17–19^, and African fossils^20–22^. Although the uniparental mtDNA and Y-DNA loci have a coalescence time of 250–150 kya, archaeological, nuclear, and genome-wide datasets suggest the possibility of a much older (500–300 kya) origin for modern humans^23–25^. The true age may rest somewhere among those two ranges.

Genome-wide coalescent analyses suggest that ancient populations began to take structure 200 kya, which led to a rift between Khoisan and non-hunter-gatherer groups (i.e., Niger-Congo, Nilo-Saharan, Afro-Asiatic) by 160 kya, followed shortly by a split between Khoisan and RFHG groups 120–100 kya^26,27^. Mitochondrial studies have reinforced this pattern of Stone Age divergences and subsequent admixture amongst rainforest^28^, and Khoisan^29,30^ hunter-gatherer groups. Much of the past 200 thousand years of human evolution has therefore been a story of population structuring and diffusion within the continent of Africa.

Our prevailing understanding of human matrilineal ancestry for nearly two decades has defined seven major African lineages or “L haplogroups.” Macrohaplogroup L is a paraphyletic group that contains all modern humans in haplogroups L0–L6^31^, except for the OOA clades M and N. We consider an L haplogroup to be any African lineage (pre-OOA) that is not nested within an existing named L haplogroup. The oldest lineage “L1” (sensu auct.) was initially rooted with an Asian sequence^10^, and was later reclassified due to paraphyly into L0, L1, and L5^32–34^. Subclades of these three oldest haplogroups reach peak frequencies in human populations thought to be outgroups to the rest of modern humans (L0d and L0k in Khoisan; L1c in Baka; L0a2b in Mbuti; L5 in Mbuti, Tshwa, Sandawe). The most widespread haplogroup in sub-Saharan Africans is L2, likely owing to the Bantu expansion, but also to a 70–50 kya climatic oscillation that drove a mass migration^35^. The remaining lineages fall into L3'4'6; i.e., L3 and its two low-frequency outgroups L4 and L6^36^. These haplogroups have an East African center of diversity, and L3 contains the only two OOA clades M and N.

We recently discovered several sequences in the private FamilyTreeDNA (FTDNA) database of full mtDNA sequences (mtFull; > 200,000 records) that cannot be classified as descendants of any of the previously described L haplogroups (see “Materials and methods”). These sequences ostensibly formed a sister clade to the ancient L5, which would be the eighth L haplogroup. This novel L7 lineage would be a major discovery, because it would be the oldest mitochondrial lineage described in two decades. Moreover, if other L7 sequences have already been mistaken as L5, this would underscore the urgency of calls to update the mtDNA tree^37,38^. The de facto resource for defining the human mtDNA topological structure is PhyloTree^31^, however it has not been updated since 2016, and its phylogenetic reconstruction methods have always been unclear. If new sequences or subclades are simply grafted onto an existing structure, then the space of possible trees is not explored, and the new context of synapomorphies is not fully considered. With just n = 50 sequences, there are (2n – 3)!! bifurcating rooted trees, or approximately the number of atoms in the known universe (~ 10^80^). Given that Phylotree v17 has > 5,000 haplogroups with many homoplastic variants, there is the possibility that a lineage as significant as L7 could go unnoticed.

In this study, we had three primary goals: (1) define the structure of L7 and its subclades; (2) estimate the timing of their origin; and (3) infer any likely population origins or migration events that led to its current distribution. Our work will shed light on the deep coalescent structure of humankind in the cradle of Africa and offer a new perspective on the most ubiquitous tool in human phylogenetics.

## Materials and methods

### Sample selection

In order to estimate the placement and age of the putative L7 clade, we selected complete mtDNA genomes to represent the backbone of the haplotype tree. These include the major subclades for L0 (L0a1, L0a2, L0d1, L0d2, L0d3, L0f., L0k), L1 (L1b, L1c1, L1c2, L1c3, L1c4), L2 (L2a, L2b, L2c, L2d, L2e), L6, L4, and L3. Inclusion of backbone sequences is important to give phylogenetic context and polarity of mutations while reconstructing L5, L7, and their relationships. To reconstruct each of these backbone branches, we selected pairs of sequences (Supplementary Table S1) representing the maximum topological distance according to the latest version of PhyloTree (v17^39^), and used HaploGrep2^40^ to infer haplogroups. We verified that publicly chosen samples did not influence the structure of L5, L7, or other parts of the tree as a consequence of any private or artifact mutations by re-running the analyses several times with multiple backbone sample sets. For L5'7, we searched extensively for any complete sequences with L5 or likely L7 mutations that could resolve this portion of the tree; we found a total of 49 L5 and 18 L7 unique sequences (Supplementary Table S2). Data sources included FTDNA private collections, Genographic Project participants opting into scientific research, the 1000 Genomes Project^41^, and the academic literature^28,30,42–52^.

### Next-generation DNA sequencing

Private collections were sequenced using the mtFull Sequence procedure in the Gene by Gene, Ltd. lab (Houston, TX). Briefly, tissue from cheek swabs were extracted using a magnetic bead procedure. Samples were LR-PCR amplified using a KAPA HiFi Hotstart PCR kit (Roche Sequencing) with the profile: 95 °C for 5 min, [98 °C for 20 s, 65 °C for 15 s, 72 °C for 3 min] × 35, 72 °C for 5 min, 4 °C hold. Amplicons were fragmented enzymatically using a QIAseq FX DNA Library kit (Qiagen), sequencing adapters were ligated, and libraries were pooled on an Illumina MiSeq lane and sequenced with 2 × 150 bp reads to 1000 × depth. We used NextGENe v2.3.1 to align raw reads and call variants. Alignment was done to the rCRS reference^9^ with a matching requirement of ≥ 12 bases and ≥ 85%, and variant calling was done according to the manufacturer's protocols. This included a 20% threshold of original reads for heteroplasmies, which were scored with IUPAC ambiguity codes.

### Sanger sequencing

We used Sanger sequencing to validate that the L7 haplogroup-defining mutations were not artifactual. Although we only needed one sequence for validation purposes, we chose three of the novel L7 sequences, one from each subclade, for robustness (see “Results”). For the sequencing reaction, proprietary primers were used with a combination of BigDye Terminator v3.1 (Applied Biosystems by Life Technologies) and custom in-house chemistries. Samples were run through capillary electrophoresis in a 3730 Genetic Analyzer (Applied Biosystems by Life Technologies), and the resulting electropherograms were scored with Sequencher 5.0 software (GeneCodes).

### Phylogenetic reconstruction and divergence dating

Each mtDNA genome was aligned to the Reconstructed Sapiens Reference Sequence^53^ using MUSCLE^54^. We removed the four most recurrent positions (152, 195, 310, 16519) to reduce the effect of homoplasy. To account for site mutation rate variation, we partitioned the data into six bins: codon position 1, 2, and 3; the hypervariable segments (HVS; HVS-I, HVS-II, and HVS-III combined); the more conserved transfer RNA (tRNA) genes; and other non-coding regions. Phylogenetic inference and divergence dating were performed with BEAST 2.5.2^55,56^. Although only SNP markers were used due to their simple model of evolution, INDELs were identified, annotated according to PhyloTree v17^39^ conventions, and used later for annotation purposes. INDELs with high recurrence or alignment ambiguity at positions 309–315, 515–524, 3105–3107, 16183–16184, and 16193 were ignored. All heteroplasmies were treated as uncertain bases. All data conversion, marker selection, and file formatting steps (e.g., Nexus) were performed in R 3.5.1^57^. PartitionFinder v1.1.1^58^ was used to select the best site model for each partition, which ranged from simple (HKY + Γ) to complex (GTR + Γ + I). After preliminary analyses using these partition-specific models, the complexity of up to 36 substitution parameters (six rates across six partitions) resulted in poor convergence. Therefore, we simplified this into an HKY + Γ model, with empirical nucleotide frequencies used for the equilibrium states. Exponential priors were used for the gamma shape parameters, and log normal priors were used for the kappa parameters of HKY. We initially compared strict and relaxed log-normal clock models, but observed no appreciable substitution rate variation across branches, based on near-zero standard deviation of the uncorrelated log-normal relaxed clock (ucld.sdev << 1; coefficient of variation 95% HPD overlapping zero), justifying the use of a strict clock.

We calibrated the strict clock with a mutation rate (μ) of 2.285 × 10^−8^ site^−1^ year^−1^, an average of mtDNA mutation rates surveyed in the literature^12,59–64^. Distributions were averaged using the distr^65^ package in R and used to specify a lognormal prior for the strict clock model. It is important to incorporate a consilience of rate estimates into the total uncertainty to offset potential bias from any one estimate^66^. We allowed the relative rates of the six partitions to be estimated and fixed to the mean overall rate. For the tree prior we selected a non-parametric Bayesian skyline model so as not to assume anything a priori about population size or tree shape through time. We performed two independent analyses of 5 × 10^7^ MCMC steps sampling every 10^3^ and used Tracer to assess a stationary distribution of posterior samples, within and between MCMC chains. We combined posterior tree samples with LogCombiner and summarized the maximum clade credibility (MCC) tree using TreeAnnotator. We repeated this process separately for just the L5 and L7 samples to estimate effective population size (N_e_) as a function of number of coalescent events through time (Bayesian Skyline analysis).

The MCC tree was summarized by collapsing any nodes with < 0.5 posterior probabilities into polytomies. We used the R package treeio^67^ to input the BEAST tree, and custom functions to collapse uncertain clades. Next, we determined which mutations were synapomorphies (i.e., defining variants) for each remaining clade. We used the data.tree package (github.com/gluc/data.tree) to convert the tree into a hierarchical data structure, and ancestral state reconstruction with the ace function in the ape package^68^. Ancestral variants defining each clade were estimated using a discrete model fitted by maximum likelihood procedure.

Inference of the best tree topology and support values of each clade differ for Bayesian and maximum likelihood inference^69,70^. Therefore, we complemented the BEAST analysis with a RAxML^71^ estimation to ensure consistent topologies, and to compare posterior probabilities with bootstrap frequencies. We ran RAxML 8.2.12 with the “-f a” algorithm to perform 10^3^ rapid bootstraps and search for the best­-scoring ML tree, under the GTRCAT model. In order to determine the outgroup for RAxML, and confirm the correct root placement by BEAST, we performed a separate BEAST analysis using 28 complete archaic mitogenomes from NCBI GenBank. All parameters were kept constant, except that only two samples from the eight L haplogroups were included, and the tree tip dates on archaic sequences were constrained using the calibrated C^14^ dates.

Some of the sequences from Gonder et al.^52^, particularly in L0, were previously identified as erroneously missing some root-defining mutations, and gaining some “phantom” (false novel) ones, due to mysterious data artifacts^43,72^. Although neither those authors nor we could find any such issues affecting the five L5/L7 sequences used in this study, we omitted them and repeated the phylogenetic analyses to ensure that the primary results were unaffected.

### Haplotype frequency maps

We obtained haplotype data from a large and representative set of studies focusing on African mtDNA to summarize frequency patterns across the continent^11,28–30,32,35,36,41–48,50–52,73–100^. We used HaploGrep2^40^ to infer haplotypes from PhyloTree v17^39^ for both full sequences and control region data. If any sequence was classified by the original authors as L5 (or L1e), we used the L5/L7 clade-defining variants (see above) to determine whether the sequence was unambiguously L5/L7, or ambiguously placed in L5'7. Definitive placement required 100% of the available SNPs for each branch or sub-branch to be positive, with no such pattern in the sister clade’s SNPs. Pie charts and heatmaps were then plotted in R v3.5.1^57^ for each major haplogroup using the ggplot2 v3.3.5^101^ and scatterpie v0.1.7 (https://github.com/GuangchuangYu/scatterpie/) packages.

### Ancestral state reconstruction of major population

We used ancestral state reconstruction to find major population patterns in the L5'7 tree. We used largest possible population groupings to capture old and distinct genetic structure: Khoisan-speakers, RFHGs, Bantu, Niger-Congo (non-Bantu), Nilo-Saharan, Afro-Asiatic, and Arabic. Several sequences had unknown population labels but did have admixture analysis results from other studies or the FTDNA database, in which case the dominant sub-Saharan population was used. We used the ace function in the ape package^68^ and a discrete model fitted by maximum likelihood procedure. Only ancestral nodes with high (> 0.9) likelihood of one population were interpreted as biologically informative.

### Ethics statement

All experiments in this study were conducted in adherence with the set of ethical principles of the Declaration of Helsinki. Ethical clearance for the study was obtained from the Pearl Institutional Review Board (protocol number: 21-GBYG-101). Informed consent was obtained from all participants.

## Results

### Phylogenetic reconstruction and divergence dating

**Figure 1:**
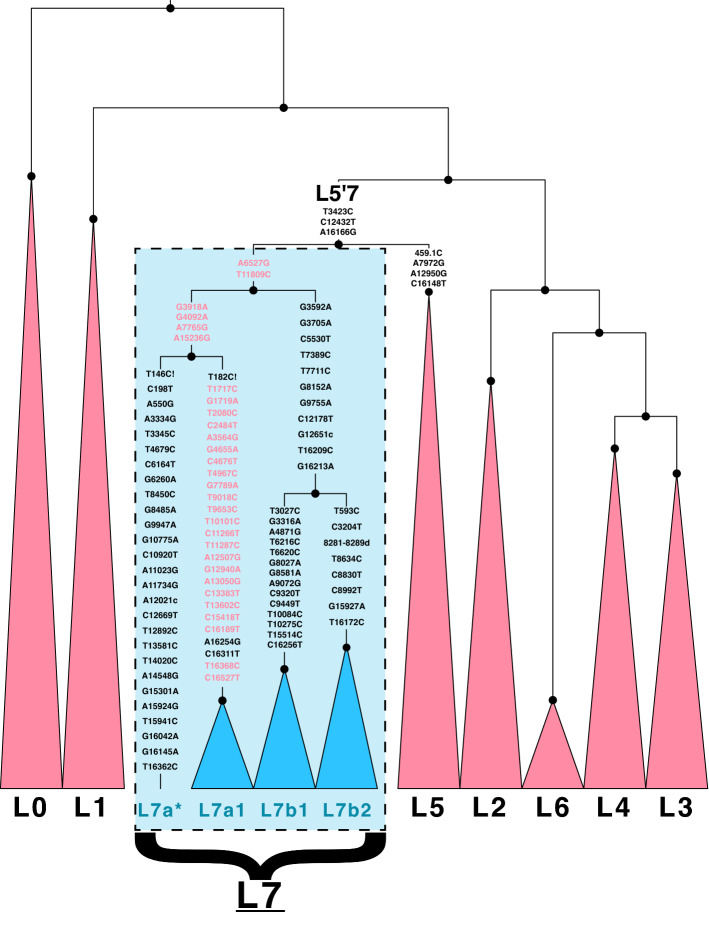
Phylogenetic Placement of New L7 Lineage. Major clades are collapsed, showing the existing (red) and newly discovered (blue) clades. Defining mutations are shown for L5'7, L5, L7, and the six major branches in L7 (L7a, L7a*, L7a1, L7b, L7b1, L7b2). Mutations formerly defining L5b2 that actually define L7 are highlighted in red.

**Figure 2:**
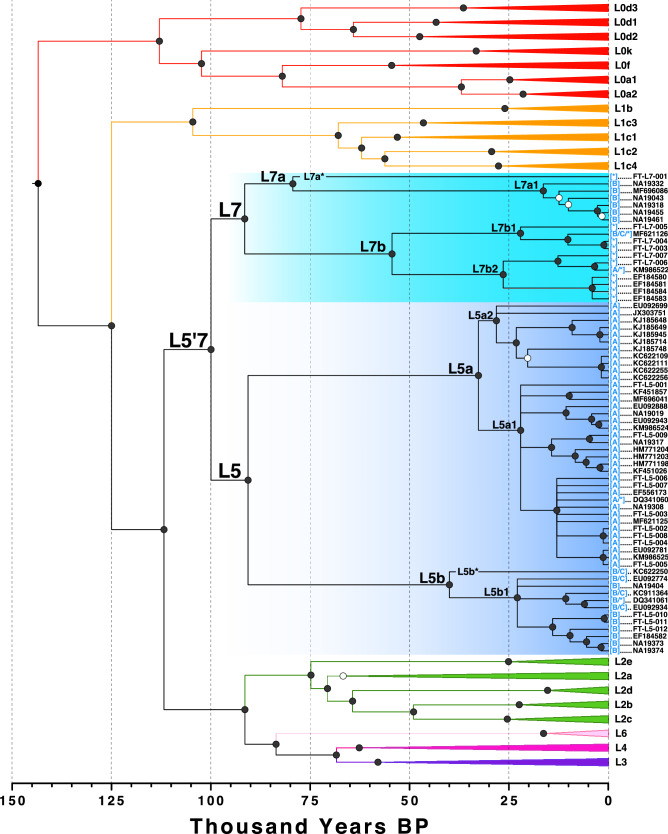
Ultrametric Time Tree. Mean divergence times estimated by BEAST with a fixed clock of $2.285\times {10}^{-8}$ substitutions site^−1^ year^−1^ are shown. L5'7 clades are highlighted in blue. Black circles denote clades with $\ge$ 0.9 posterior probability, open circles denote clades with $\ge$ 0.5 but $<$ 0.9 posterior probability, and polytomies include any clades $<$ 0.5 posterior probability. Previous haplogroup assignments (in square brackets) are denoted as follows: * = L5, A = L5a, B = L5b, C = L5c (which is an undescribed haplogroup).

Eighteen unique sequences were found to have the unique pattern: derived for the three L5 synapomorphic mutations T3423C, C12432T, and A16166G, yet negative, or ancestral for the other four (459.1C, A7972G, A12950G, C16148T), and further derived for two additional SNPs A6527G and T11809C (Fig. 1). They were also found to share the retromutation at position 195, but we excluded this hypervariable mutation from our analysis (see “Materials and methods”). Our BEAST and RAxML analyses recovered a topology that confidently splits these 18 samples into a new clade (L7) that is reciprocally monophyletic with L5, united by the new parent clade L5'7 (Fig. 2, Supplementary Figs. S1–S4; Supplementary Tables S3, S4). All newly sequenced samples were sequenced to a mean depth of 1167× across all sites, and 1402× at these relevant sites.

Three L7 samples in the academic literature have previously been assigned to subclades of L5 based on PhyloTree v17: KM986522 (L5a1b;^51^), MF696086 (L5b2;^45,102^), and MF621126 (L5b2a;^50^). However, Vyas et al.^51^ indicated an uncertain placement (“L2'3'4'5'6”), and Cabrera et al.^50^ correctly suggested their Sudanese sequence MF621126 formed a sister group to known L5 which they termed “L5c.” We elevate this clade to “L7” for two reasons. (1) The samples currently assigned to L5a and L5b are not valid (reciprocally monophyletic) groups since samples in L5b2 are actually sister lineages to all of L5 (i.e., they are L7). Hence, the current nomenclature must change (see Supplementary Table S5 for a mapping between new and previous clades). Elevating L7 re-establishes monophyly and integrity for L5, L5a, and L5b. (2) L7 is even older than L5, making it the third oldest haplogroup after L0 and L1 (Fig. 2; Supplementary Table S3). The newly described L5'7 split is the seventh oldest divergence in the tree at 100.0 [111.9–87.7] kya. We note that the name L7 has been proposed once before to clade “L4g”^46^, however that was never adopted elsewhere in the literature or PhyloTree database.

In addition to the three L7 samples previously assigned to L5 subclades, we found four L7 Tanzanian samples assigned generically to L5^52^ or “L2'3'4'5'6a1a”^102^, five samples in the 1000 Genomes Project^41^, and six unique samples from the private FTDNA database and the Genographic Project^103^. Sanger sequencing independently confirmed the variant calls in samples from three major L7 subclades: FT-L7-002 (L7a*), FT-L7-004 (L7b1), and FT-L7-006 (L7b2). We found no effect on any parent or sister clades after experimentally removing the samples from Gonder et al.^52^. Preliminary BEAST analyses using a relaxed log-normal clock showed little rate variation across branches (ucld.sdev = 0.208; coefficient of variation = 0.21) justifying the assumption of a strict clock.

One sequence within the L7a clade (L7a*) shares a most recent common ancestor approximately 79 kya with its sister clade L7a1 (Table 1). Two first cousins were found to share this identical sequence: FT-L7-001, and FT-L7-002 (unused in phylogenetic analyses), both individuals of primarily European descent with South African matrilineal ancestry. The L7a* lineage is therefore the oldest autapomorphic sequence currently known in the human mtDNA tree, with 27 derived SNPs (Table 1; Fig. 2).

**Table 1:** Mean divergence estimates and mutations for major L5'7 subclades.

Clade	Parent	Mean years bp (95% HPD)	Stem Length (years)	Posterior Prob	Mutations
L5'7	L2'3'4'5'6'7	99,960 (111,909–87,700)	11,833	1.000	T3423C C12432T A16166G
L7	L5'7	91,476 (104,169–78,614)	8,485	1.000	A6527G T11809C
L7a	L7	79,365 (93,730–65,749)	12,111	1.000	G3918A G4092A A7765G A15236G
L7a*	L7a	0	79,365	–	T146C! C198T A550G A3334G T3345C T4679C C6164T G6260A T8450C G8485A G9947A G10775A C10920T A11023G A11734G A12021c C12669T T12892C T13581C T14020C A14548G G15301A A15924G T15941C G16042A G16145A T16362C
L7a1	L7a	16,333 (23,350–9,683)	63,032	1.000	T182C! (573.XC) T1717C G1719A T2080C C2484T A3564G G4655A C4676T T4967C G7789A T9018C T9653C T10101C C11266T T11287C A12507G G12940A A13050G C13383T T13602C C15418T C16189T A16254G C16311T T16368C C16527T
L7b	L7	54,409 (67,795–41,528)	37,067	1.000	G3592A G3705A C5530T T7389C T7711C G8152A G9755A C12178T G12651c T16209C G16213A
L7b1	L7b	22,081 (30,890–14,007)	32,328	1.000	T3027C G3316A A4871G T6216C T6620C G8027A G8581A A9072G C9320T C9449T T10084C T10275C T15514C C16256T
L7b2	L7b	26,435 (36,356–16,851)	27,974	1.000	T593C C3204T 8281-8289d T8634C C8830T C8992T G15927A T16172C
L5	L5'7	90,636 (103,698–77,497)	9,324	1.000	459.1C A7972G A12950G C16148T
L5a	L5	32,665 (39,890–25,777)	57,972	1.000	455.1 T G709A A851G T1822C C5111T G5147A A5656G G6182A T6297C A7424G G8155A A8188G C8582T G9305A G9329A T11025C C11881T G12236A A13105G! A13722G T14212C C14239T T14581C G14905A T14971C G15217A G15884A A16183c C16355T T16362C
L5a1	L5a	22,045 (27,833–16,613)	10,619	1.000	455.2 T G930A C4496T C8754T
L5a2	L5a	28,133 (34,677–21,847)	4,532	0.998	C527T G8856A
L5b	L5	39,969 (51,726–28,445)	50,667	1.000	A3720G A9809c T10493C T11701C T12188C A12546t T12714C A12810G T13569C T13830C C16111T A16254G C16360T
L5b1	L5b	22,885 (30,103–16,074)	17,085	1.000	A249d C535T C2417g T3027C A4976G C5213T C16311T
L5b*	L5b	0	39,969	-	C2380T T4233C A4529G T4907C C6173a C8829T T8937C G9966A T10045C T11287C G12406A A15442G

**Figure 3:**
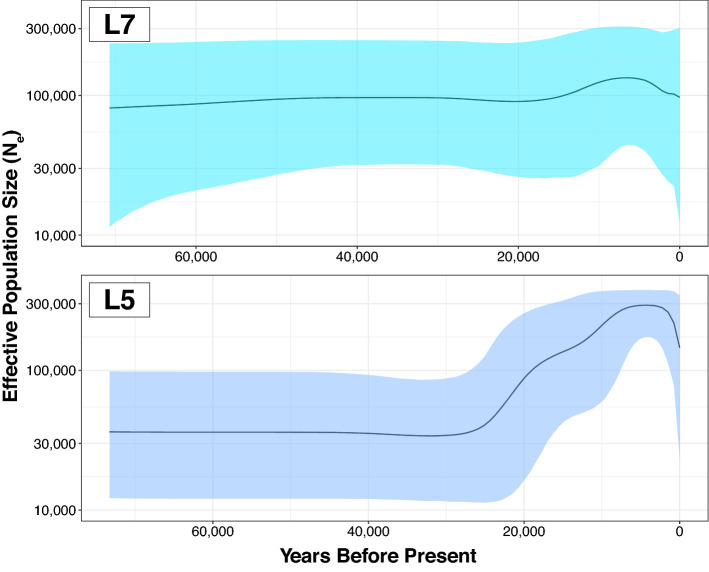
Bayesian Skyline Plot. Mean N_e_ as a function of coalescent event probability is shown through time for clades L7 and L5, shaded by upper and lower 95% HPD intervals.

Three major subclades of L7 were discovered in addition to the L7a* singleton branch: L7a1 dated to 16.3 [23.4–9.7] kya, L7b1 dated to 22.1 [30.9–14.0] kya, and L7b2 dated to 26.4 [36.4–16.9] kya. BEAST and RAxML both yielded consistent topologies for all major clades (Supplementary Figs. S1, S2). The major L5 subclades L5a1, L5a2, and L5b1 were also found to have mean divergence time estimates between ca. 22 and 28 kya, coinciding with an estimated increase in population size (Fig. 3). Our BEAST analysis of ancient mitogenomes from Neanderthals, Denisovans, and *Homo heidelbergensis* (Sima de los Huesos sample; SDLH) confirmed L0 as the outgroup in modern human mtDNA and estimated a divergence time of ~ 725 kya for their common hominid ancestor (Supplementary Fig. S3; Supplementary Table S6). The inferred introgression event from pre-modern humans into Neanderthals was dated to ~ 380 kya, which is consistent with earlier work on the SDLH sample^5,104,105^.

### Haplotype frequency maps

**Figure 4:**
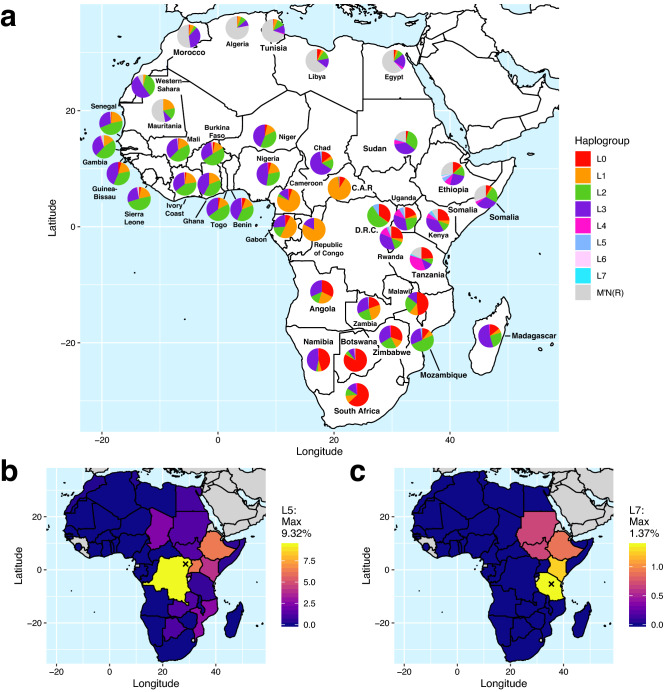
Distribution of Haplotypes by Population. (**a**) Frequencies of each major haplogroup by African country, with color codes identical to Fig. 2. D.R.C. = Democratic Republic of the Congo, C.A.R. = Central African Republic; (**b**) Heatmap of L5 frequency by country normalized to maximum; (**c**) Heatmap of L7 frequency by country normalized to maximum. For b–c, Countries shaded in gray have no data, and an ‘ × ’ marks the locality with highest frequency (Mbuti population in b, Sandawe population in c).

African frequencies of the eight L haplogroups reach their peak diversity in the eastern Rift Valley countries of Ethiopia, Kenya, and Tanzania (Fig. 4, Supplementary Fig. S5, S6). We accumulated frequency data from 11,089 African samples across 46 studies (Supplementary Table S7). Minority clades such as L3i, L3x, L4, L5, L6, and L7 reach their highest frequencies in these countries, although generally at less than 10% of the local population (except for L4: 35% in Tanzania). After reclassifying all known L5'7 sequences into either L5 or L7 wherever the available markers permitted, we found that L5 reaches peak frequency (9.32%) in the Mbuti population of eastern Democratic Republic of the Congo (D.R.C.), and L7 (1.37%) in the Sandawe population of Tanzania (Fig. 4b,c).

### Ancestral state reconstruction of major population

**Figure 5:**
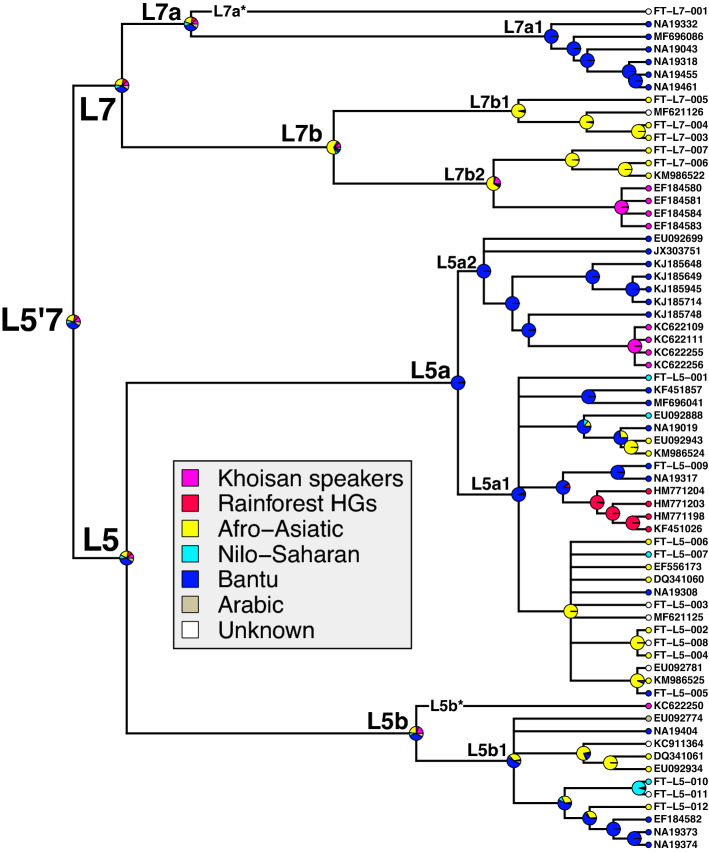
Ancestral Reconstruction of Population Groups. Major population groupings for each sequence are shown as small colored circles at the tips of the tree. Each ancestral pie chart represents the likelihood that node derives from each population.

Recent subclades of L5 and L7 tend to share common population groupings, although older subclades have far more uncertain population origins (Fig. 5). We used a population classification scheme that attempts to group the 221 recorded African populations in our dataset into seven major population lineages (Supplementary Table S8). For example, we found that L7a1 is comprised entirely of Bantu (Luhya and Swahili) ethnic groups in Kenya. In contrast, the four L7b1 samples are associated with Afro-Asiatic groups in Ethiopia and outside of Africa in the Middle East: Cushitic and Semitic admixture for two FTDNA customers from Dubai and Ethiopia, Jordanian and Palestinian history in a third. The fourth sample with Sudanese background could not be unambiguously grouped. The multiple occurrences of L7b1 outside of Africa are peculiar and would require further sample discovery on either side of the Red Sea to help establish geographic origin for the subclade. Similarly, L7b2 samples from FTDNA have Ethiopian origins and Semitic-Cushitic admixture in two cases, or Yemeni origins in a third case.

Importantly, the four Khoisan-speaking Sandawe people form their own ~ 4 kya subclade within the ~ 26 kya clade L7b2. An analogous pattern of primarily Bantu or Afro-Asiatic clades is seen in the L5a and L5b topologies, which circumscribe smaller subclades of foraging populations. For example, an ~ 8 kya subclade of Mbuti RFHG people is found in L5a1, and a ~ 1.7 kya subclade of Khoisan Tshwa people is found in L5a2 (Fig. 5). The L5b* lineage is only known from a single sequence shared by two Khoisan Shua individuals in Botswana, whose most recent common ancestor with L5b1 lived ~ 40 kya.

## Discussion

### Discovery of an ancient lineage

Discovery of an eighth L haplogroup that diverged ~ 100 kya and has a coalescence time of ~ 90 kya is an exciting but unexpectedly late advance in human mtDNA phylogenetics after amassing thousands of sequences for 40 years. The first clades to be assigned “haplogroups” were ordered by their discovery, first A–G in Asia and the Americas^106,107^, H–K in Europe^108^, and only one haplogroup L for the most diverse continent of Africa^10^. Due to incorrect rooting with an Asian sequence, the initial L haplogroups L1 and L2 were rerooted to achieve monophyly in L1, resulting in the first five L haplogroups L0, L1, L5, L2, and L3 containing OOA-subclades M and N^32–34^. Thus, in early papers the L5 lineage was originally described as “L1e”^109^. Rare eastern African L haplogroups L4 and L6 were the last two to be described^36^. The ancient divergences in L7, its novel parent group L5'7 (coalesced ~ 100 kya), and the oldest singleton branch L7a* (diverged ~ 80 kya), may shed light on early demographic events of modern humans in East Africa.

### Middle stone age expansions in early humans

The earliest modern human population divergences are thought to include: (1) foraging peoples from the ancestors of non-foraging peoples by ~ 160 kya, and (2) Khoisan from other foragers such as RFHGs and Hadza and Sandawe ancestors by ~ 120–100 kya^26,27^. Subsequent divisions between Niger-Congo, Nilo-Saharan, and Afro-Asiatic lineages and between eastern and western branches of Khoisan and RFHGs occurred less than 55 kya. Modern-day populations such as Hadza and Sandawe illustrate the complexity that ensued: after a ~ 88 kya split from each other, they admixed with Afro-Asiatic groups such as Omotic and Cushitic, and with the Khoisan from whom they likely derive their click-language^110^. Gradual population growth followed by several major demographic expansions in the Middle Stone Age ~ 75–55 kya^35,111–113^ were the likely catalysts for advancing new terrestrial and marine foraging abilities, microlithic technology, novel pigments in art, and possibly even syntactic language^114–119^. Although most African populations retain a genetic signal of expansion from ~ 70 kya or as early as ~ 110 kya in East African Nilotic groups, serial bottlenecks have largely erased this expansion signal from hunter-gatherer groups^120^.

Such oscillating periods of population divergence followed by major expansion events with admixture between groups may reflect episodic climatic change across the continent. During glacial maxima, reduced forestation has resulted from increased aridity and desertification, restricting humans to lakeshores, river margins, oceansides, and highlands of Kenya and Ethiopia with persistent canopy cover^121^. Such oases of habitat may have been important refugia during the arid OIS 6 period from ~ 200–125 kya, if woodland habitat was favored by humans as a source of water, food, and protection from heat and predation. Archaeological and climatic evidence suggests an improvement after this period, however East and tropical Central Africa show inverse population abundances, especially from 130–60 kya, with an increase in Central Africa and a decrease in East Africa^122^. Changing monsoonal patterns caused wet periods in East Africa during 145–120, 110–95, 80–65, and 55–50 kya, and simultaneous arid periods in Central Africa, including a “megadrought” from 115 to 90 kya^122–124^. Volcanic activity caused by a series of caldera collapses along the Eastern African Rift System also likely made the region uninhabitable for segments of time^122^. Thus, asynchronous pulses of climatic and tectonic instability^125^ likely forced human dispersals back and forth across the African continent during much of prehistory.

The L7 and L5 haplogroups diversified ~ 90 kya during this complex period of climatically driven divergence events in East Africa. That coincides with the end of favorably wet conditions in the East African cradle of mtDNA diversity, and the end of a western superdrought, which may have pressured a slight western and southward expansion into the eastern Congo Basin and Great Lakes region of western Tanzania. Other major lineages such as L2 also arose in West Africa at this time before later returning eastward^35^. Both haplogroups L7 and L5 retain minor subclades that are purely foraging groups: Sandawe people of Tanzania in the case of L7; and both Tshwa/Shua Khoisan of Botswana and east RFHGs of the D.R.C. for L5 (Fig. 5). Each haplogroup also reaches its peak frequency within those populations (Fig. 4b,c). This pattern may hint at the original autochthonous populations that founded these two clades. If Khoisan, RFHGs, and Sandawe foraging populations had relatively small sizes compared to the groups they have recently intermixed with, genetic drift would be expected to drive higher rates of lineage extinction. Previous studies have surmised this process to explain high haplotype frequencies in many foraging groups: L0d and L0k across Khoisan groups^29,43^; L5 in Tshwa and Shua Khoisan^30^; L3d in Damara Khoisan^30^; L1c1a and L1c4 in Baka RFHGs^126^; L0a2b, L5, L2a2, L2a4 in Mbuti RFHGs^28^; L4 in Sandawe and Hadza^78^; and now L7 (formerly “L5”) in Sandawe^52^.

Population history is rarely represented by any single genetic locus such as mtDNA, for many reasons: (1) phylogenies are diverging (bifurcating or multifurcating) whereas populations are usually reticulating since most populations are products of admixture between ancestral populations; (2) genetic drift causes lineages within smaller populations to be lost faster; and (3) incomplete lineage sorting (fueled by drift) can prevent any single-locus phylogeny from tracking the correct branching order of populations. However, even if a large clade has mixed population identity due to drift or gene flow, a younger subclade may still provide useful population ancestry information if most subclade members are from one population. For example, Khoisan ancestors are thought to be the outgroup to other modern humans, yet L0 (the mtDNA outgroup) is found among many African populations; however, the two subclades L0d and L0k are comprised almost entirely (82% and 83% respectively) of Khoisan-speakers. Typically, this is interpreted to reinforce a correspondence between Khoisan and L0^30,43,52^. The high population diversity of other L0 subclades may represent ancient admixture with those groups, and the specificity of Khoisan-speakers in L0d/L0k may represent the drift within this shrinking population.

Shrinking populations of autochthonous foragers during the past 20 kya may explain why the majority of L5'7 descendants today are primarily Afro-Asiatic (e.g., Cushitic Ethiopian, Ethiopian Jewish, or Yemenite), or eastern Bantu (e.g., Luhya). For example, eastern RFHGs who retain the highest L5 percentage gradually experienced a 40% reduction in effective population size (N_e_) starting 20 kya^28^. A simultaneous seven-fold increase in pre-farming populations suggests that non-foraging populations expanded and possibly subsumed many rare haplogroups by female gene flow. This would explain the long stem lengths for L5a, L5b, L7a, and L7b, with more recent coalescence times of ~ 20 kya (Fig. 2), coinciding with a higher growth rate (Fig. 3). The mtDNA and ethno-linguistic diversity in East Africa is unparalleled in the continent, largely due to the coexistence of ancient linguistic and genetic groups such as Omotic, Cushitic, Semitic, Nilotic, and Chadic^73,88^. Admixture with these expanding groups may have preserved the exceedingly rare L7 lineage (maximum frequency 1.37%; Fig. 4c). West African Bantu farmers expanded eastward and southward 5–2 kya and assimilated with most local genetic and linguistic groups, leading to their having 15–25% ancestry from west RFHGs, Khoisan, and Afro-Asiatic farmers^47,127,128^. This second wave of admixture may be the source of the Bantu ancestry in L7a1 and many subclades of L5. The ~ 80 kya relict sequence L7a* from a primarily European woman of South African matrilineal descent is very intriguing. It exposes the process of lineage extirpation, either due to tens of thousands of years of low N_e_, or a recent bottleneck (or both). Given that there is < 1% detectable sub-Saharan ancestry in the individual, and the next closest sequence is 80 kya removed, it is imprudent to speculate about the history of this lineage. With time and intensive sampling in southeastern Africa, it is possible more L7a* sequences will be discovered and will help resolve the mystery.

### Need to revise the human mtDNA tree

The L7 sequence remained undiscovered partly due to its rarity (< 2% of any population). Only 18 unique full sequences are currently known, and the two synapomorphic SNPs for L7 are in the coding region, thus making them invisible across numerous control region datasets. However, 12 of the 18 full sequences were already publicly available. PhyloTree^39^ and other semi-static reference trees are also in need of continual re-estimation to avoid overlooking important topological changes such as the L7 clade. So called “phylogenetic placement” methods that use a fixed tree topology as prior information to assign unknown sequences have their place^129–131^, particularly when the sequence does not differ significantly from known sequences, or in metagenomics. However, any new sequence added to a tree alters the context of ancestral and derived states, which is why heuristic algorithms such as TBR or NNI are normally employed in modern phylogenetics to explore optimal tree space^132^.

Often in human mtDNA phylogenetic studies, maximum parsimony (MP) methods are used^34,42,53,79,88^, which do indeed explore tree space heuristically. However, MP uses the optimality criterion of fewest changes (minimum homoplasy), which is not often true with increasing timespans and faster mutation rates of hypervariable base positions. A common issue with MP is long branch attraction, whereby unrelated branches that experienced large amounts of evolutionary change are erroneously placed together due to shared mutations^133,134^. Independently derived mutations can be shared for many reasons in mtDNA, including rapid mutation rate causing convergence by chance, but also selection for mito-nuclear compatibility, which often sorts heteroplasmies according to the nuclear genetic background^135,136^. Maximum likelihood and Bayesian methods are more ideal for phylogenetic reconstruction of nucleotide sequences because they explicitly model site rate variation and between-lineage heterotachy, although in theory all methods should converge on the same answer if given informative data^137^.

What is needed now is a periodic re-estimation of the mtDNA tree that incorporates all complete sequences worldwide, and then accurately classifies unknown sequences using the novel reference tree. Such a project has been undertaken recently with a total of 2,243 sequences and MP^37^, but this falls short of the hundreds of thousands of existing haplotypes that would undoubtedly form myriad new haplogroups. The recently initiated Million Mito Project^138^ combines data from multiple sources to re-estimate the phylogenetic Tree of Womankind, restructuring, redefining and annotating the branches using more than a quarter of a million current samples, with the goal of incorporating one million full mtDNA sequences.

## Conclusions

We have discovered the third oldest L haplogroup fully 40 years after Anderson^9^ unveiled the first human mtDNA sequence. Hidden within L7 is the oldest known singleton branch of the tree. This exciting twist to the matrilineal story of humanity is undoubtedly not the last, as many unique haplotypes lie hidden and waiting for discovery. Future work should corroborate our preliminary inference that the Sandawe were an autochthonous source of L7 ancestry by resampling that and related populations in the Dodoma region of Tanzania. Although we attempted to identify control region sequences that are L7 using downstream L7a and L7b SNPs, many possible L7 may still elude us, and therefore future studies should fully sequence any such L5'7 samples. We encourage the human mtDNA research community to seek out other ambiguously placed haplotypes based on PhyloTree as potentially new L haplogroups, or other rare or misplaced haplogroups. A global initiative to catalogue and re-estimate the phylogeny for all full mtDNA sequences, such as the Million Mito Project^138^, should aim to more efficiently track the evolving shape of the Tree of Womankind.

## Supplementary Information

Supplementary Information 1.

Supplementary Information 2.

## Data Availability

The seven novel L7 complete mitochondrial sequences produced in this study are available in the NCBI GenBank repository, under accession numbers ON156774–ON156780. The ancillary L5 sequences produced during the current study are not publicly available due to FamilyTreeDNA privacy terms, but upon reasonable request to the corresponding author, permission can be sought from the participants.
