# Supplementary material for: African mitochondrial haplogroup L7: a 100,000-year-old maternal human lineage discovered through reassessment and new sequencing
Source: Sci Rep. 2022 Jun 24;12:10747. doi: 10.1038/s41598-022-13856-0 (PMC9232647; doi:10.1038/s41598-022-13856-0)
Supplement: Supplementary file 1 — Supplementary Information 1. [file 41598_2022_13856_MOESM1_ESM.pdf]

Supplementary Figures S1–S6

African Mitochondrial Haplogroup L7: A 100,000-Year-Old Maternal Human Lineage Discovered Through Reassessment and New Sequencing

Paul A. Maier[1,2,6*], Göran Runfeldt[1,6], Roberta J. Estes[3,1,6], Miguel G. Vilar[4,5,1,6]

[1] FamilyTreeDNA, Gene by Gene, 1445 N Loop W, Houston, TX 77008, USA

[2] Department of Evolution, Ecology, and Organismal Biology, University of California Riverside, 900 University Ave, Riverside, CA 92521, USA

[3] DNAeXplained, www.dna-explained.com, USA

[4] Department of Anthropology, University of Maryland, 7999 Regents Dr, College Park, MD 20740, USA

[5] Genographic Project, National Geographic Society, 1145 17th St NW, Washington, DC 20036, USA

[6] Million Mito Project, USA

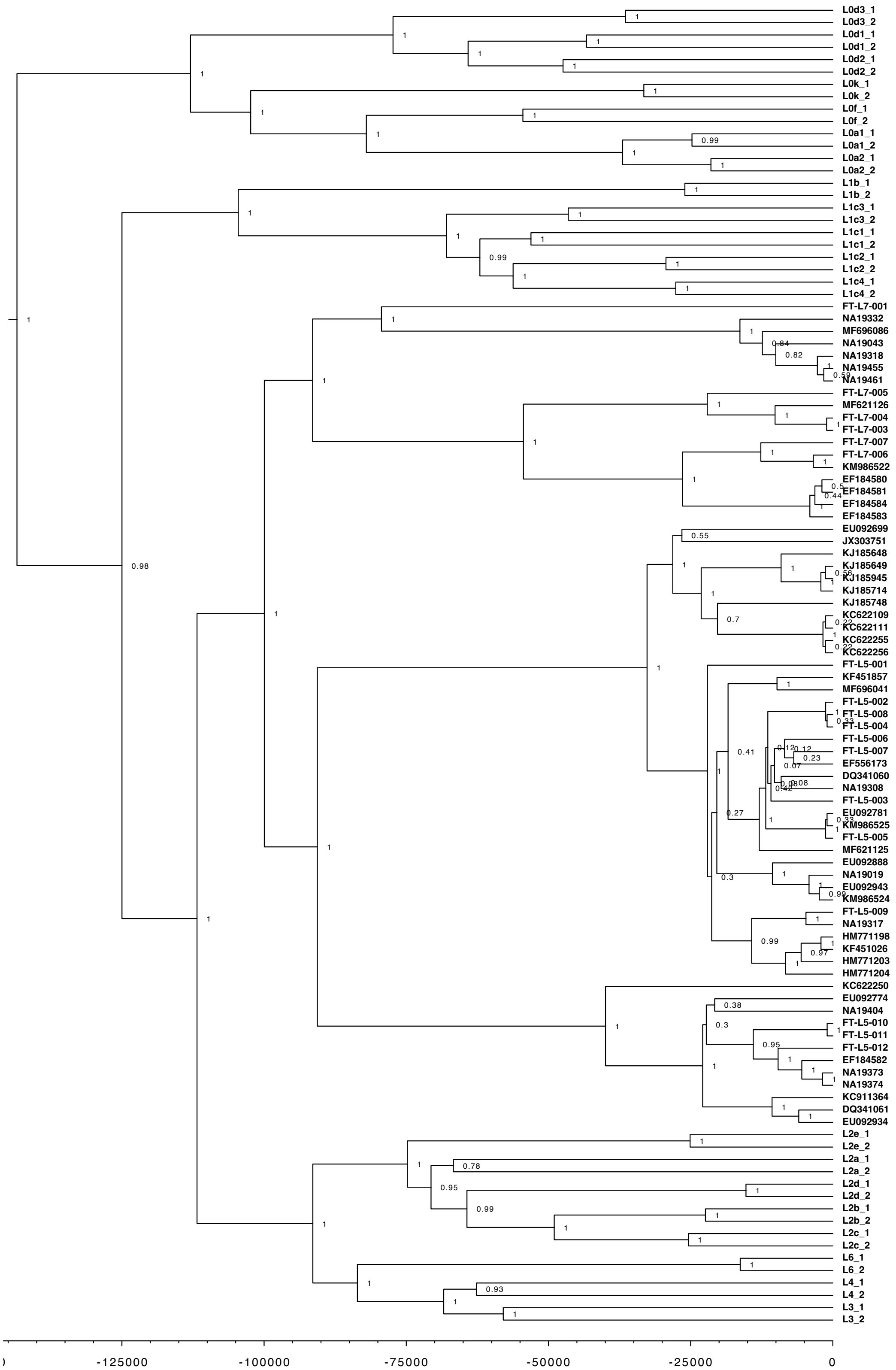

Figure S1. Ultrametric BEAST Tree. Original (uncollapsed) Bayesian tree topology is shown with posterior probabilities at each node. Samples are described in Tables S2 (L5'7) and S1 (all other haplogroups).

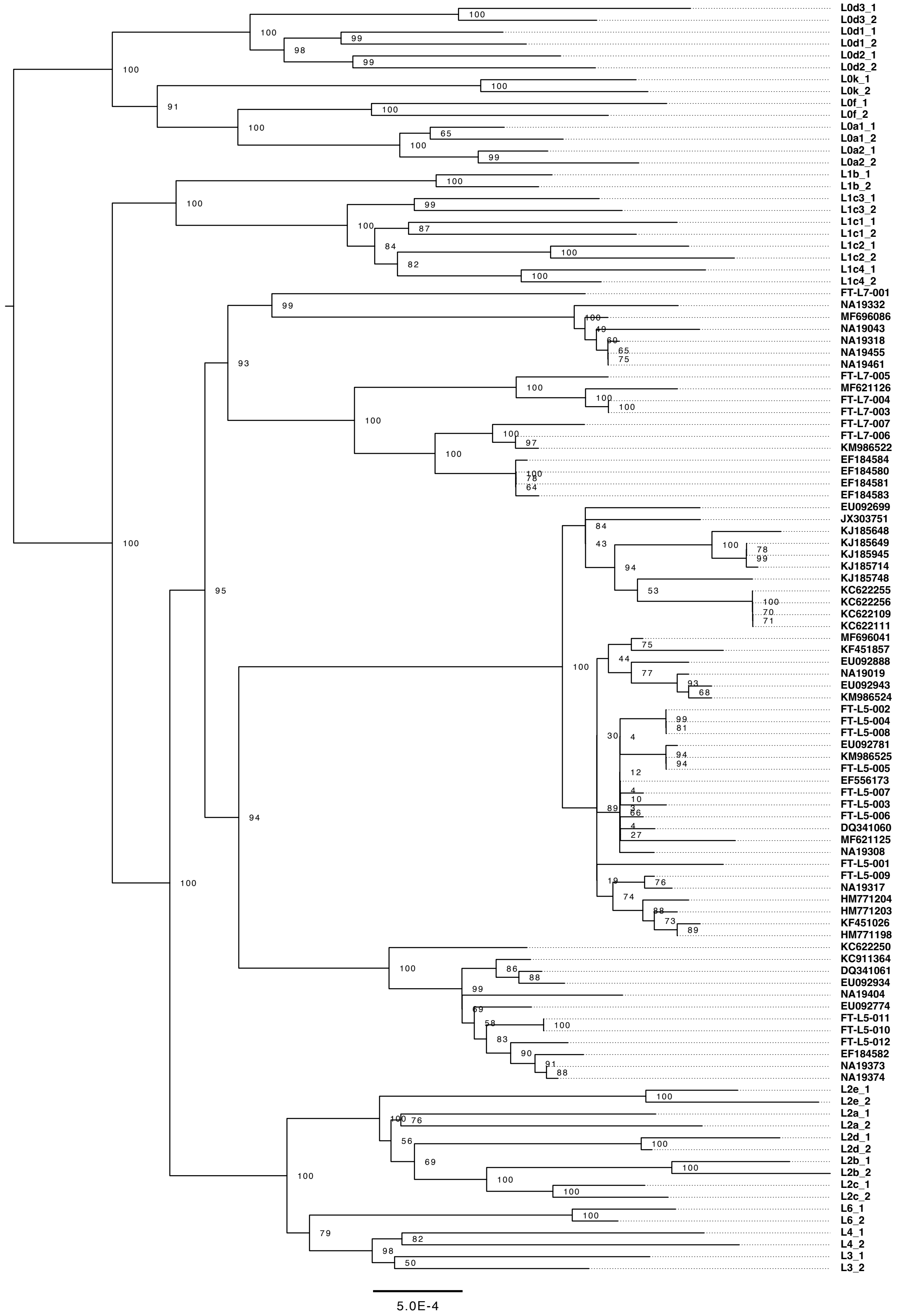

Figure S2. RAxML Tree. Original (uncollapsed) maximum likelihood tree topology is shown with frequencies from 10^3 rapid bootstrap replicates. Samples are described in Tables S2 (L5'7) and S1 (all other haplogroups).

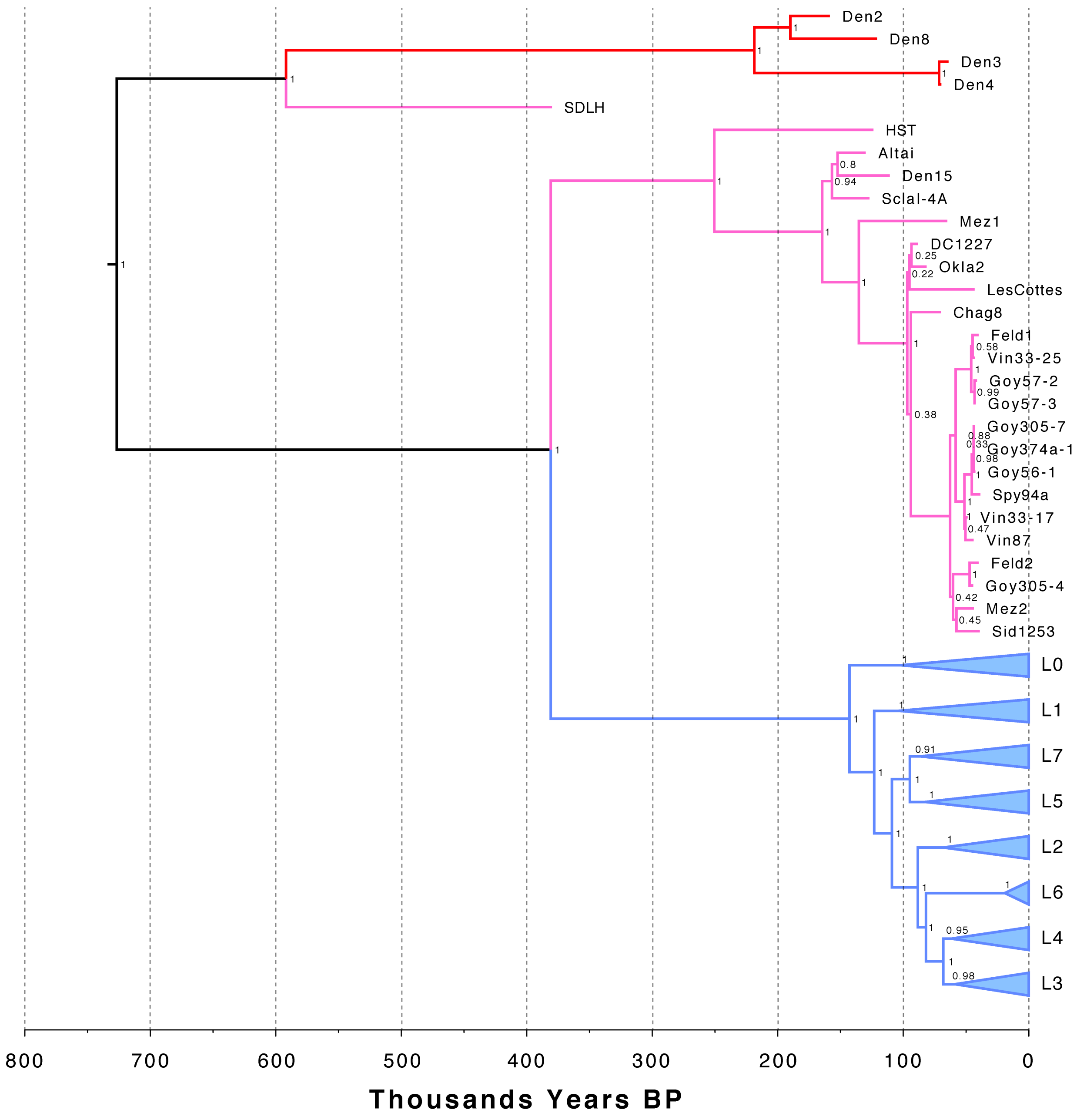

Figure S3. Archaic Human Outgroup Analysis. Ultrametric BEAST tree including two pairs of samples for each of the eight L haplogroups, and 28 archaic (Neanderthal and Denisovan) samples from NCBI GenBank. Tip dates are derived from calibrated C^{14} dates. Ancient sample accessions and tip dates are listed in Table S6. Modern sample accessions for L5'7 and other haplogroups are listed in Tables S2 and S1, respectively (noted as: "Used for archaic tree").

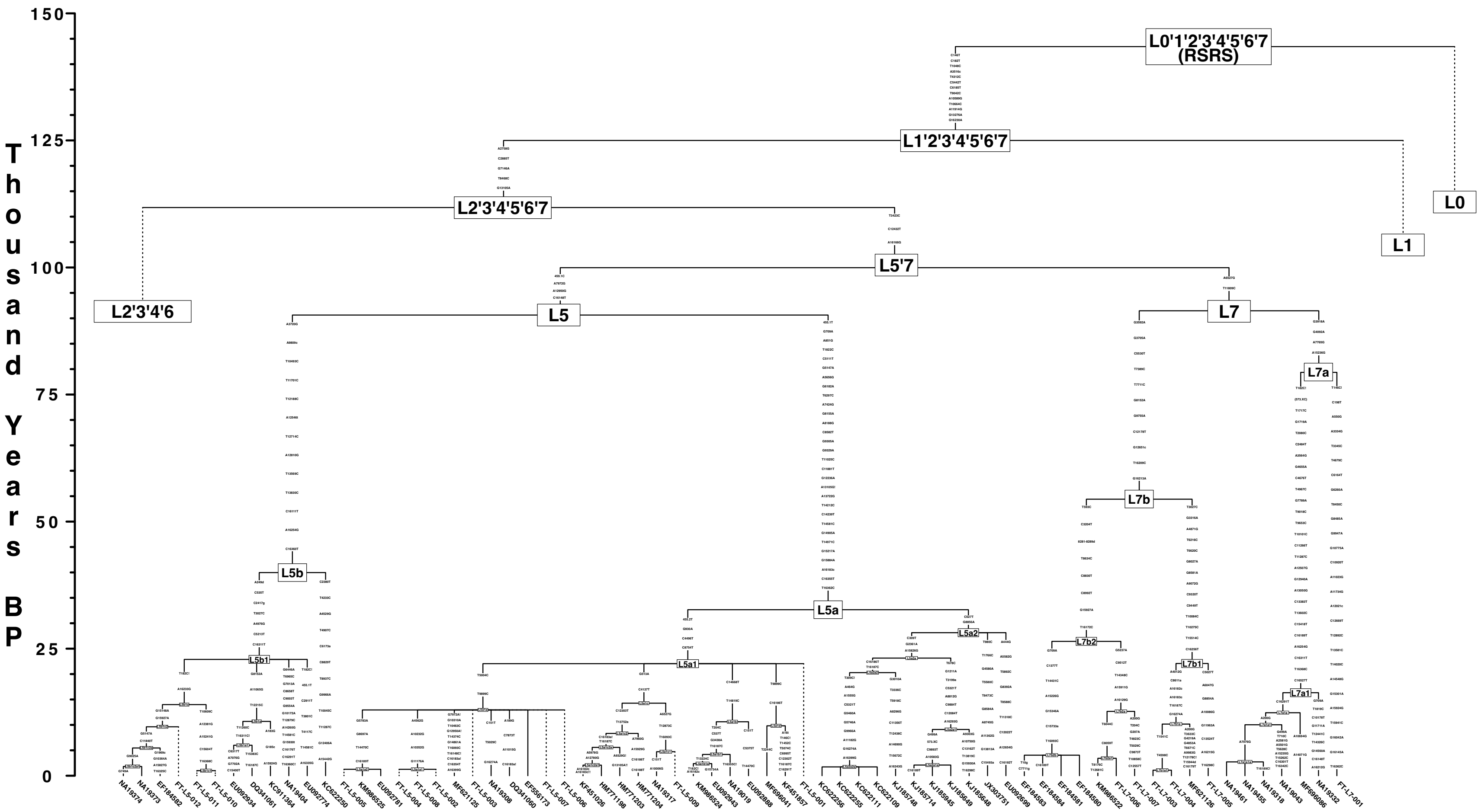

Figure S4. Full Mutational Map of L5'7 Phylogeny. Mutations for all subclades described in Table S4 are shown, except for branches denoted with dotted lines. L0'1'2'3'4'5'6'7 is synonymous with "Mitochondrial Eve." Samples listed in Table S2.

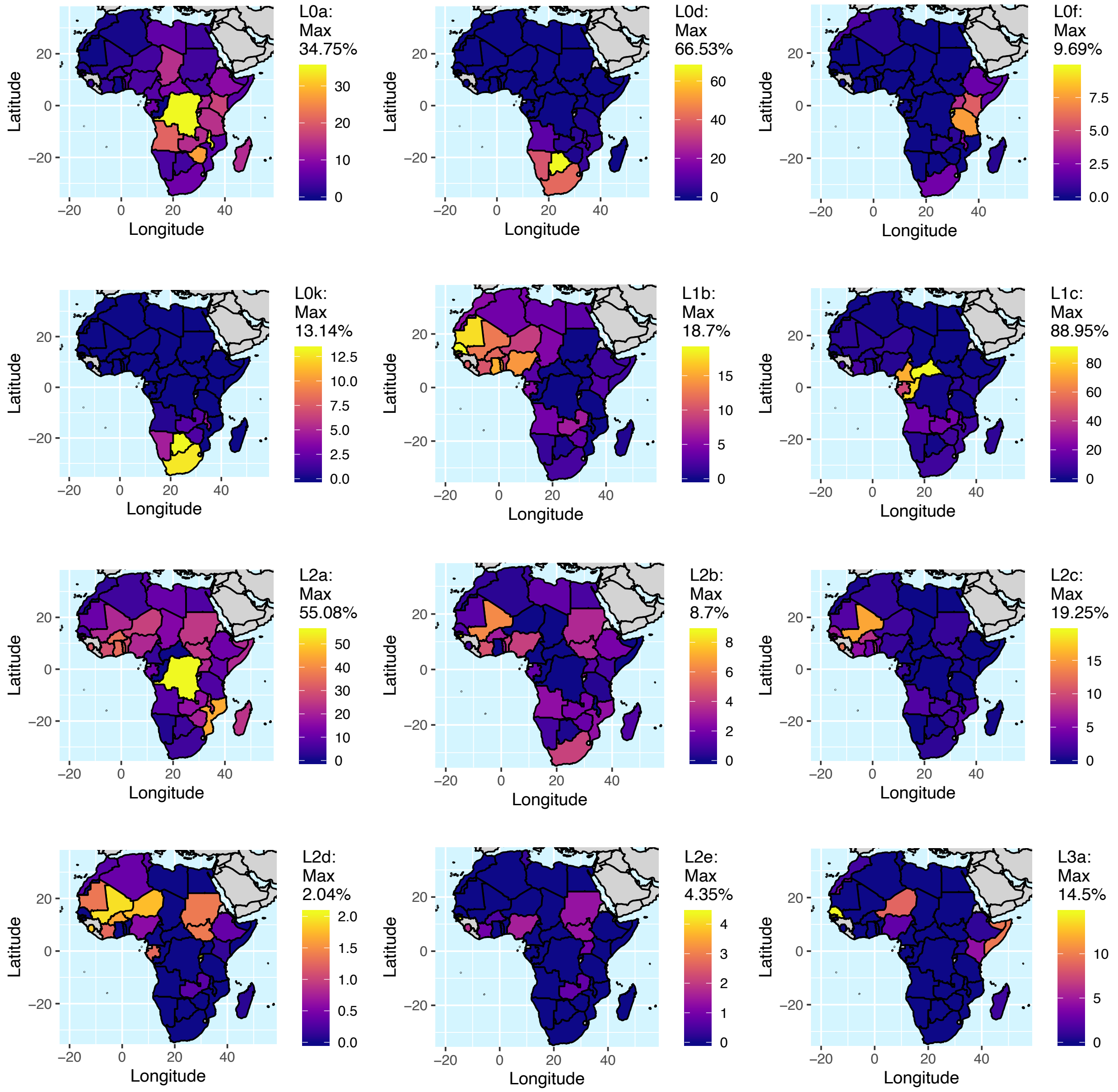

Figure S5. Distribution of Haplotypes by Population. Heatmap of haplogroup frequency by country normalized to maximum. Only haplogroups with sample sizes greater than 20 were included. From left to right, top to bottom: L0a, L0d, L0f, L0k, L1b, L1c, L2a, L2b, L2c, L2d, L2e, L3a.

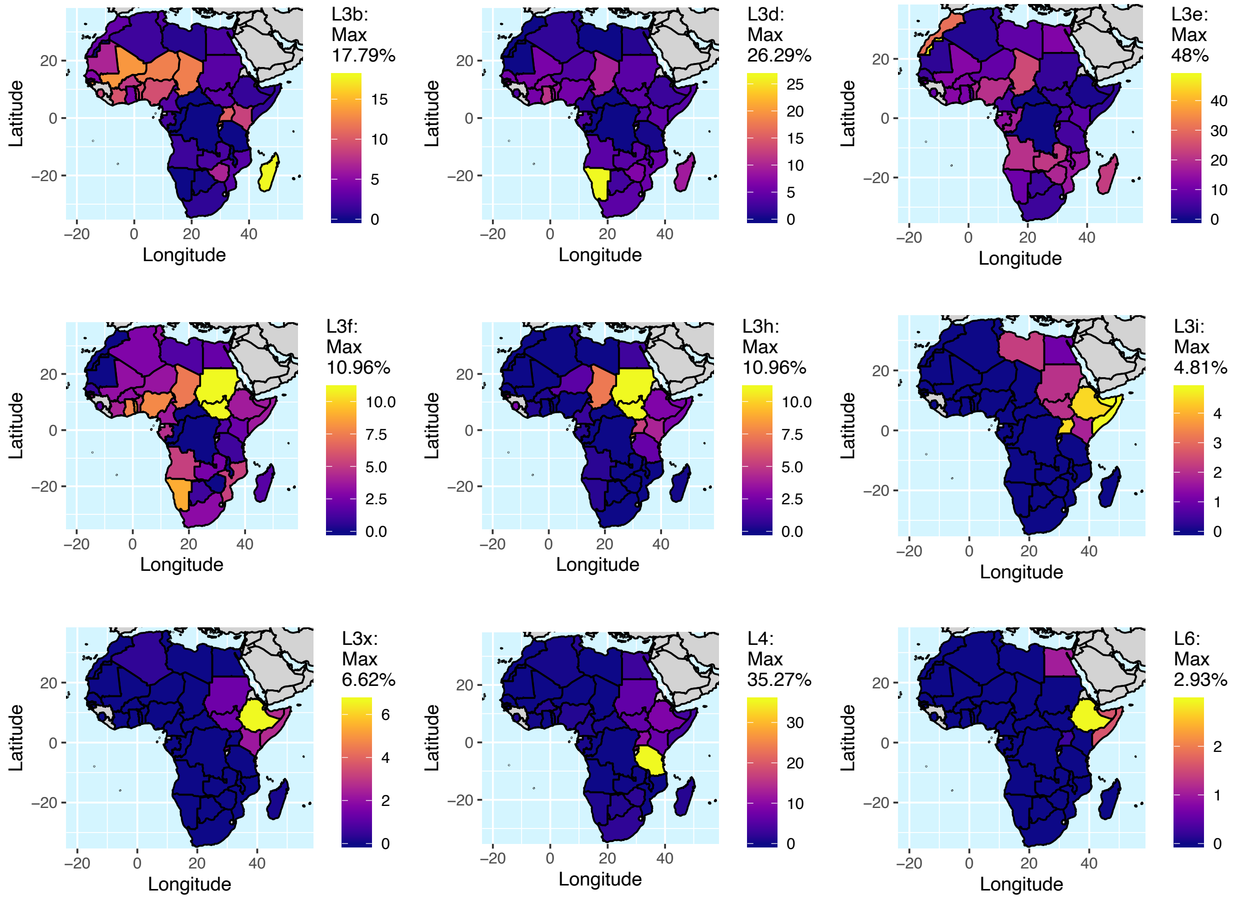

Figure S6. Distribution of Haplotypes by Population. Heatmap of haplogroup frequency by country normalized to maximum. Only haplogroups with sample sizes greater than 20 were included. From left to right, top to bottom: L3b, L3d, L3e, L3f, L3h, L3i, L3x, L4, L6.
